# Association Between 20-Year Trajectories of Nonoccupational Physical Activity From Midlife to Old Age and Biomarkers of Cardiovascular Disease: A 20-Year Longitudinal Study of British Men

**DOI:** 10.1093/aje/kwy157

**Published:** 2018-08-14

**Authors:** Daniel Aggio, Efstathios Papachristou, Olia Papacosta, Lucy T Lennon, Sarah Ash, Peter H Whincup, S Goya Wannamethee, Barbara J Jefferis

**Affiliations:** 1 Department of Primary Care and Population Health, University College London, London, United Kingdom; 2 University College London Physical Activity Research Group, London, United Kingdom; 3 Department of Psychology and Human Development, University College London, London, United Kingdom; 4 Population Health Research Institute, St George’s University of London, London, United Kingdom

**Keywords:** aging, cardiovascular biomarkers, cardiovascular disease, inflammation, physical activity

## Abstract

The trajectories of physical activity (PA) from midlife into old age and their associations with established and novel cardiovascular disease (CVD) risk factors in later life remain unclear. This study examined associations between 20-year nonoccupational PA trajectories and a range of CVD biomarkers at ages 60–79 years. We used data from a sample of 3,331 men (mean baseline age = 50.2 ± 5.8 years) recruited in 1978–1980, with follow-up after 12, 16, and 20 years, reporting habitual nonoccupational PA at each wave. At the 20-year follow-up, surviving men attended a physical examination and provided a fasting blood sample. Group-based trajectory modeling was used to identify trajectories. Adjusted regression analyses examined the association between trajectory-group membership and several cardiometabolic, cardiac, and inflammatory markers at follow-up. Three distinct 20-year trajectories were identified: low/decreasing (21.3%), light/stable (51.8%), and moderate/increasing (27.0%). Compared with the low/decreasing group, membership in the light/stable and moderate/increasing trajectory groups was associated with a more favorable cardiometabolic profile and lower levels of inflammation and endothelial dysfunction. Although following a moderate-increasing PA trajectory was most favorable, more modest but sustained doses of PA into old age may be sufficient to lower CVD risk.

There is clear evidence that regular physical activity (PA) is associated with a lower risk of cardiovascular disease (CVD) morbidity and mortality (1, 2). The transition to old age is a crucial life stage when future PA levels and the subsequent risk of developing CVD in later life are determined (3, 4).

Previous studies examining the association between change in PA and CVD biomarkers in old age are limited by relatively short follow-up and few repeated measurements of PA, resulting in important PA fluctuations being missed (5–10). Several of these studies have shown that PA has cumulative effects on CVD risk factors, with sustained high PA being most strongly related with lower levels of established metabolic risk factors, inflammation, endothelial dysfunction, and cardiac injury in old age (5–7, 9, 10). Only a few prospective studies with long-term follow-up have used more than 2 or 3 repeated measures of PA to examine associations with CVD biomarkers in old age (11).

Existing studies typically use clinical cutpoints to collapse PA data into a binary or ternary format to distinguish between those who maintain or change their PA levels (9–11). This conventional method for identifying trajectories assumes these clinically relevant trajectory groups truly exist. By contrast, group-based trajectory modeling (GBTM) is a data-driven approach that has the ability “to identify rather than assume distinctive groups of trajectories” (12, p. 139), thus describing the true underlying trajectories of behavior change over time (13). Previous studies have used this technique to identify trajectories of PA into old age (14–16), but to our knowledge it has not yet been used to examine associations with CVD biomarkers. Longitudinal studies using more repeated measures of PA and data-driven techniques are required to understand how patterns of PA into old age are associated with established and novel CVD risk factors. The aim of this study was therefore to explore associations between 20-year trajectories of nonoccupational PA from midlife to old age, identified using GBTM, and CVD biomarkers in old age in men without preexisting CVD or diabetes at baseline.

## METHODS

### Participants

Data were drawn from the British Regional Heart Study, an ongoing prospective cohort study following 7,735 men (aged 40–59 years at baseline) recruited from primary care practices in 24 towns in Great Britain between 1978 and 1980 (17). Men completed a lifestyle and medical history questionnaire at baseline and at 12, 16, and 20 years, and they attended physical examinations at baseline and 20-year follow-up. Participants provided informed written consent to the investigation. Ethical approval was obtained from the National Research Ethics Service Committee London.

### Measures

#### CVD biomarkers

At the 20-year follow-up, men provided a fasting blood sample, which was used for laboratory tests of cholesterol (total, high-density lipoprotein, and low-density lipoprotein), triglycerides, glycated hemoglobin, factor VIII, plasma levels of tissue plasminogen activator (tPA) antigen, D-dimer, von Willebrand factor, C-reactive protein (CRP), interleukin-6 (IL-6), *N*-terminal pro-brain natriuretic peptide, and high-sensitivity cardiac troponin T. Nurses measured waist circumference, blood pressure, lung function (forced expiratory volume in 1 second), and height and weight, from which body mass index (BMI) was calculated (as weight (kg)/height (m)^2^). Hypertension was defined as a systolic blood pressure of ≥160 mm Hg, diastolic blood pressure ≥100 mm Hg, or use of antihypertensive medication (18). Further information on measurement techniques is provided in Web Appendix 1 (available at https://academic.oup.com/aje).

#### Self-reported PA

At all follow-up examinations, participants reported their usual PA. Questions included time spent on all forms of walking, recreational activities (such as recreational walking, gardening, chores, and do-it-yourself activities), and sports/exercise. The same questions and response options were administered at each wave. Responses were scored based on the intensity and frequency of the activity (19, 20). For example, making no journeys by foot was scored as 0 and >90 minutes/weekday was scored as 5. Scores were also heavily weighted for vigorous activities. For example, playing sports 4–7 times a month was given a score of 8. Scores for each item were summed together to give a total PA index. The original scoring system has been reported in detail elsewhere (21). The total PA index was then used to group men into 6 categories (0–5); 0: inactive; 1: occasional (regular walking or recreational activity only); 2: light (more frequent recreational activities, sporting exercise less than once a week, or regular walking plus some recreational activity); 3: moderate (cycling, very frequent weekend recreational activities plus regular walking, or sporting activity once a week); 4: moderately vigorous (sporting activity at least once a week or frequent cycling, plus frequent recreational activities or walking, or frequent sporting activities only); or 5: vigorous (very frequent sporting exercise or frequent sporting exercise plus other recreational activities). The 6-point total PA score used for the analysis has been validated against heart rate, forced expiratory volume in 1 second (21), and device-measured PA (22).

#### Covariates

A range of sociodemographic characteristics, health conditions, and lifestyle behaviors were reported in the questionnaire. These included age, employment status (employed or not in employment), current or longest held occupation (manual or nonmanual), marital status (single, married, or widowed/divorced), number of children (none or ≥1), doctor-diagnosed health conditions (including arthritis, bronchitis, and high blood pressure), other health problems (including breathlessness and chest pain on exertion), BMI (normal weight: <25.0; or overweight/obese: ≥25.0), smoking status (current/recent former-smoker or nonsmoker/long-term former-smoker (>15 years)), alcohol consumption (none, occasional (<1 drink/week), light (1–15 drinks/week), moderate (16–42 drinks/week), or heavy (>42 drinks/week)), region of residence (Scotland, North, Midlands, or South), and weekly breakfast cereal consumption (none, occasional (1–2 times/week), or regular (>3 times/week)). Men also reported doctor-diagnosed CVD events, including stroke (with symptoms lasting >24 hours), heart attack, myocardial infarction, coronary thrombosis and angina, and diabetes mellitus. In addition, medication use (warfarin, blood pressure-lowering medication, and lipid-lowering medication) was recorded at 20-year follow-up.

### Statistical analysis

#### Identifying PA trajectory groups

GBTM was used to identify PA trajectories over the 4 time points in men without stroke, coronary heart disease, or diabetes at baseline. Analyses were conducted using the Stata (StataCorp LLC, College Station, Texas) TRAJ plugin (23), which applies finite mixture models and maximum likelihood estimation to identify groups of individuals that follow similar patterns of behavior over time. To identify the optimal number of trajectory groups, models with 2–5 groups were tested and compared using goodness-of-fit criteria. The best-fitting model was selected based on highest (i.e., least negative) Bayesian information criterion, the log Bayes factor (2 × Δ Bayesian information criterion), sufficient trajectory group sizes (i.e., at least 5% of participants in each trajectory group), close agreement between the estimated probability of group membership and the actual proportion of the sample assigned to that group, posterior probabilities of >0.70, and odds of correct classification based on posterior probabilities exceeding 5 (13). For each subject, the model provides the probability of belonging to each of the identified trajectory groups and assigns them to the trajectory group based on the highest probability. Models for identifying these groups were simultaneously adjusted for baseline age, occupational class, marital status, number of children, region of residence, diagnosed health conditions (arthritis, bronchitis, and high blood pressure), BMI, smoking status, alcohol consumption, and breakfast cereal consumption as well as time-varying covariates, measured at each wave, including number of CVD events and employment status. To determine the shape of each trajectory, the level of the polynomial function for each group was reduced, starting with quadratic, until each growth parameter estimate was statistically significant (*P* < 0.05).

#### Regression analyses

Once the optimal shape and number of trajectories were determined, descriptive characteristics and CVD biomarkers were calculated for each trajectory group. Linear regression was then used to estimate the association between PA trajectory-group membership and CVD biomarkers at the 20-year follow-up. Because many of the men were being treated for high blood pressure by the time of the 20-year follow-up, logistic regression was used to estimate the odds of having hypertension (including taking antihypertensive medication) according to trajectory-group membership. Regression models were adjusted for current age, smoking status, alcohol consumption, waist circumference (except when waist circumference was the outcome), occupational class, region of residence, and use of lipid-lowering and antihypertensive medication (except when hypertension was the outcome). Models for tPA antigen, D-dimer, von Willebrand factor, and factor VIII were also adjusted for warfarin use. Insulin, glycated hemoglobin, IL-6, CRP, D-dimer, *N*-terminal pro-brain natriuretic peptide, high-sensitivity cardiac troponin T, and glucose were log transformed because they were positively skewed. Trends across trajectory groups were tested entering the trajectory group variable as a continuous variable.

#### Additional and sensitivity analyses

To examine whether current PA is a stronger predictor of CVD risk factors than PA trajectories, we included both the PA trajectories and the 6-point total PA score at the 20-year follow-up in the models. In addition, to explore whether GBTM yielded additional insights compared with a more conventional approach of categorizing trajectories of PA, the trajectories were instead defined manually using a binary PA variable across 3 time points (baseline, 12-year follow-up, and 20-year follow-up). At each of the 3 time points, men were classified as active (1) if they had at least “light” activity or inactive (0) for inactive or occasional activity. Men were allocated to 1 of 8 possible trajectories, which are indicated by combinations of zeros and ones (1, active; 0, inactive). For example, 0-0-0 represents inactivity at all periods, while 1-0-0 indicates being active at baseline only. Regression analyses were repeated with the manually derived trajectories as the exposure. Further, to minimize the effects of reverse causality, whereby those who were already predisposed to develop CVD or diabetes during the trajectory period go on to participate less in PA, regression models were refitted excluding men with CVD or diabetes at the 20-year follow-up. To determine the effects of adiposity on the estimates, regression models were refitted without adjusting for waist circumference. Finally, regression models were refitted after applying the probabilities of trajectory group membership as weights, giving more weight to individuals with a higher probability of belonging to a particular group. The same adjustments were made in these additional models as described in the main regression analyses.

## RESULTS

Of the 7,735 men invited, men with <3 PA assessments (*n* = 2,752) or a diagnosis of stroke (*n* = 52), coronary heart disease (*n* = 292), or diabetes (*n* = 156) at baseline were excluded. A further 1,465 men were excluded due to incomplete biomarker and covariate data, leaving a final sample of 3,331 men (flow diagram, Web Figure 1). Compared with the final sample, men who were excluded were less active at baseline (total PA score, 1.9 vs. 2.4; *P* < 0.001) and more likely to come from manual occupations (71.4% vs. 52.4%; *P* < 0.001), be older (52.4 vs. 48.7 years; *P* < 0.001), and suffer from a range of health conditions, such as breathlessness (12.2% vs. 2.8%; *P* < 0.001), overweight/obesity (55.2% vs. 52.5%; *P* < 0.001), arthritis (12.6% vs. 8.1%; *P* < 0.001), and bronchitis (23.8% vs. 13.9%, *P* < 0.001).

**Figure 1.**
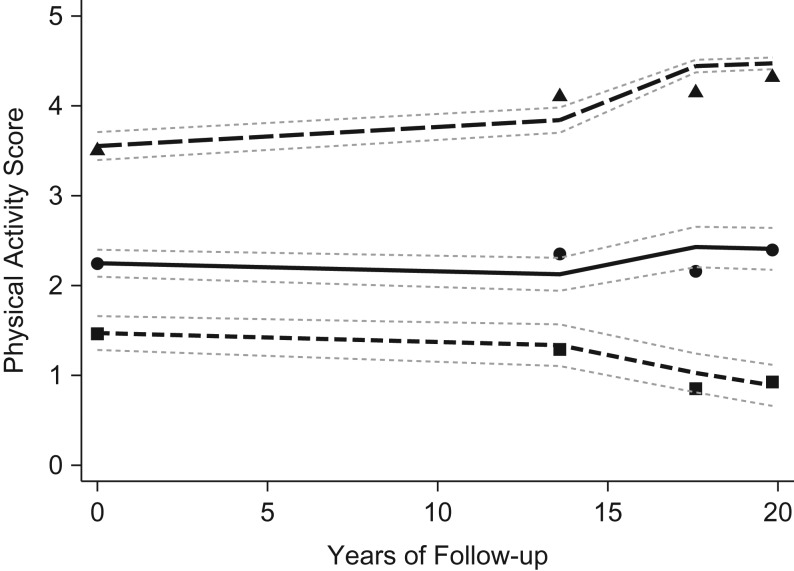
Physical activity trajectories from midlife to old age (*n* = 3,331; aged 58–81 years at follow-up), British Regional Heart Study, 1978–2000. The small dashes (with squares) represent the plotted trajectory curve for the low/decreasing group (21.3%), the solid line (with circles) represents the light/stable group (51.8%), and the large dashes (with triangles) represent the moderate/increasing group (27.0%). The plotted points show the mean physical activity score and 95% confidence intervals at each time point for each trajectory group.

As previously described in this cohort (14), 3 trajectory groups of PA over the course of 20 years best described the data: low/decreasing (21.3%), light/stable (51.8%), and moderate/increasing (27.0%) (see Figure 1). Results from the model selection process are provided in Web Tables 1 and 2. Sociodemographic characteristics, lifestyle behaviors, health status, and CVD biomarkers are presented according to trajectory group in Table 1.

**Table 1.** Characteristics of 3,331 Men at 20-Year Follow-up According to Physical Activity Trajectory Group, British Regional Heart Study, 1978–2000

	Physical Activity Trajectory Group											
Characteristic	Low/Decreasing (*n* = 708)				Light/Stable (*n* = 1,725)				Moderate/Increasing (*n* = 898)				Total (*n* = 3,331)			
	No.	%	Mean (SD)	Median (IQR)	No.	%	Mean (SD)	Median (IQR)	No.	%	Mean (SD)	Median (IQR)	No.	%	Mean (SD)	Median (IQR)
Age			69.4 (5.7)				68.5 (5.5)				67.7 (5.1)				68.5 (5.5)	
Manual occupational class	428	60.5			966	56.0			350	39.0			1,744	52.4		
Current/recent former-smoker	276	39.0			395	22.9			158	17.6			829	24.9		
At least moderate alcohol consumption^a^	129	18.2			340	19.7			172	19.2			641	19.2		
Resident in southern England	189	26.7			608	35.3			339	37.8			1,136	34.1		
Taking antihypertensive medication	298	42.1			520	30.1			215	23.9			1,033	31.0		
Taking lipid-lowering medication	54	7.6			113	6.6			63	7.0			230	6.9		
Taking warfarin	26	3.7			50	2.9			18	2.0			94	2.8		
HDL, mmol/L			1.3 (0.3)				1.3 (0.3)				1.4 (0.3)				1.3 (0.3)	
LDL, mmol/L			3.8 (1.0)				4.0 (1.0)				3.9 (0.9)				3.9 (1.0)	
Total cholesterol, mmol/L			5.9 (1.0)				6.0 (1.1)				6.0 (1.1)				6.0 (1.1)	
Triglycerides, mmol/L				1.7 (1.2)				1.6 (1.0)				1.5 (1.0)				1.6 (1.0)
Glucose, mmol/L				1.7 (0.2)				1.7 (0.1)				1.7 (0.1)				1.7 (0.2)
Insulin, μ/mL				9.6 (8.0)				8.1 (6.0)				7.5 (5.3)				8.2 (6.4)
HbA1C, mmol/L				5.0 (0.8)				4.8 (0.7)				4.8 (0.7)				4.8 (0.8)
Hypertension^b^	424	59.9			865	50.1			401	44.7			1,690	50.7		
FEV_1_, L^c^			234.5 (67.4)				260.2 (63.4)				276.5 (61.1)				259.1 (65.3)	
Waist circumference, cm			99.9 (10.9)				96.5 (10.0)				95.3 (9.5)				96.9 (10.2)	
IL-6, pg/mL				2.8 (2.5)				2.2 (1.8)				2.0 (1.5)				2.2 (1.9)
Factor VIII, IU/dL			138.2 (31.6)				130.8 (31.0)				127.5 (29.5)				131.5 (31.0)	
von Willebrand factor, IU/dL			147.9 (48.0)				137.5 (44.5)				133.4 (43.6)				138.6 (45.3)	
tPA antigen, ng/mL			12.3 (4.9)				10.8 (4.2)				10.2 (4.0)				11.0 (4.4)	
CRP, mg/L				2.3 (4.0)				1.4 (2.4)				1.2 (1.9)				1.5 (2.6)
D-dimer, ng/mL				86.0 (106.0)				75.0 (75.0)				68.0 (60.0)				74 (77.0)
Hs-TnT, pg/mL				12.6 (8.2)				11.3 (7.0)				11.5 (6.3)				11.6 (7.1)
NT-proBNP, pg/mL^d^				108.0 (208.0)				87.0 (133.0)				76.5 (108.0)				88.0 (141.0)

Abbreviations: CRP, c-reactive protein; FEV_1_, forced expiratory volume in 1 second; HbA1C, glycated hemoglobin; HDL, high-density lipoprotein; Hs-TnT, high-sensitivity cardiac troponin T; IL-6, interleukin-6; IQR, interquartile range; LDL, low-density lipoprotein; NT-proBNP, *N*-terminal pro-brain natriuretic peptide; SD, standard deviation; tPA, tissue plasminogen activator.

^a^ Moderate alcohol consumption was defined as 16–42 drinks/week.

^b^ Hypertension was defined as a systolic blood pressure of ≥160 mm Hg, diastolic blood pressure ≥100 mm Hg, or use of antihypertensive medication.

^c^ Standardized for height by multiplying FEV_1_ by the square of the mean population height (meters) divided by each participant’s height.

^d^ Data were missing for an additional 194 men (*n* = 3,137).

Men in the low/decreasing group were older, less likely to come from the south of England, and more likely to come from manual occupations and be a recent or current smoker (*P* < 0.05). Regression analyses revealed that men in the light/stable and moderate/increasing groups had a more favorable CVD biomarker profile than men in the low/decreasing group (see Table 2). This included lower levels of insulin, glycated hemoglobin, blood glucose, inflammatory markers (IL-6 and CRP), and hemostatic factors (von Willebrand factor, factor VIII, tPA antigen, and D-dimer); a smaller waist circumference; and higher lung function and reduced odds of hypertension. Men in the moderate/increasing group also had lower levels of triglycerides. The magnitude of the associations was typically stronger for the moderate/increasing group, and there was a significant linear trend across trajectory groups. In addition, the light/stable group had significantly lower levels of the cardiac marker high-sensitivity cardiac troponin T in comparison with the low/decreasing group.

**Table 2.** Adjusted Association^a^ Between 20-Year Physical Activity Trajectories and Cardiovascular Markers at 20-Year Follow-up From Logistic or Linear Regression Models (*n* = 3,331 Men, Aged 58–81 Years at Follow-up), British Regional Heart Study, 1978–2000

Outcome Measure	Physical Activity Trajectory Group^b^				*P* for Trend
	Light/Stable		Moderate/Increasing		
	B Coefficient	95% CI	B Coefficient	95% CI	
Metabolic markers					
HDL, mmol/L^c^	−0.03	−0.06, 0.00	0.01	−0.02, 0.05	0.304
LDL, mmol/L^c^	0.09	0.00, 0.17	0.04	−0.06, 0.14	0.550
Total cholesterol, mmol/L^c^	0.04	−0.05, 0.13	−0.01	−0.12, 0.10	0.798
Triglycerides, mmol/L^c^	−0.03	−0.06, 0.01	−0.06	−0.11, −0.02	0.005
Hypertension^d^	0.78	0.64, 0.94	0.66	0.53, 0.81	
Glucose, mmol/L^c^	−0.02	−0.04, −0.01	−0.03	−0.05, −0.01	0.005
Insulin, μ/mL^c^	−0.08	−0.13, −0.03	−0.11	−0.17, −0.06	0.001
HbA1C, mmol/L^c,e^	−0.02	−0.03, −0.01	−0.02	−0.04, −0.01	0.014
FEV_1_, L^c,f^	13.92	8.64, 19.19	22.21	16.09, 28.33	<0.001
Waist circumference, cm^c^	−3.25	−4.13, −2.36	−4.22	−5.24, −3.19	<0.001
Inflammatory/hemostatic markers					
IL-6, pg/mL^c,e^	−0.12	−0.18, −0.07	−0.18	−0.24, −0.11	<0.001
CRP, mg/L^c,e^	−0.21	−0.30, −0.11	−0.27	−0.38, −0.17	<0.001
Factor VIII, IU/dL^c,g^	−5.01	−7.72, −2.31	−5.72	−8.86, −2.58	0.001
von Willebrand factor, IU/dL^c,g^	−5.73	−9.65, −1.81	−6.42	−10.97, −1.88	0.009
tPA antigen, ng/mL^c,g^	−0.69	−1.05, −0.33	−1.05	−1.46, −0.63	<0.001
D-dimer, ng/mL^c,e,g^	−0.10	−0.16, −0.03	−0.11	−0.19, −0.03	0.010
Cardiac markers					
Hs-TnT, pg/mL^c,e^	−0.07	−0.11, −0.03	−0.03	−0.08, 0.02	0.303
NT-proBNP, pg/mL^c,e,h^	−0.07	−0.17, 0.02	−0.06	−0.17, 0.06	0.381

Abbreviations: CI, confidence interval; CRP, c-reactive protein; FEV_1_, forced expiratory volume in 1 second; HbA1C, glycated hemoglobin; HDL, high-density lipoprotein; Hs-TnT, high-sensitivity cardiac troponin T; IL-6, interleukin-6; LDL, low-density lipoprotein; NT-proBNP, *N*-terminal pro-brain natriuretic peptide; tPA, tissue plasminogen activator.

^a^ All models adjusted for age, occupational class, region of residence, smoking status, alcohol consumption, waist circumference (except where waist circumference was the outcome, when models adjusted for all other factors except waist circumference), and lipid-lowering medication.

^b^ Low/decreasing group served as the reference group.

^c^ Additionally adjusted for blood pressure-lowering medication.

^d^ Presented as an odds ratio and 95% confidence interval. Hypertension was defined as a systolic blood pressure of ≥160 mm Hg, diastolic blood pressure ≥100 mm Hg, or use of antihypertensive medication.

^e^ Log transformed.

^f^ FEV_1_ was standardized for height by multiplying FEV_1_ by the square of the mean population height (meters) divided by each participant’s height.

^g^ Additionally adjusted for warfarin.

^h^ Data were missing for an additional 194 men (*n* = 3,137).

Associations were also examined after including both PA trajectory and current PA score (20-year follow-up) in the models (Web Table 3). Inclusion of the current PA score attenuated the association between trajectories of PA and CVD biomarkers. Associations between trajectories and IL-6, von Willebrand factor, and D-dimer were largely attenuated and no longer significant. Associations with CRP were also markedly attenuated to nonsignificance in the moderate increasing group. Associations with metabolic markers were only marginally attenuated, many remaining significant apart from insulin and hypertension, which were borderline significant. Additional analyses using manually derived trajectories revealed similar associations with persistent activity proving optimal (Table 3). Persistent inactivity (0-0-0) was most consistently associated with the least favorable outcome. Associations with lung function, IL-6, CRP, tPA antigen, and D-dimer were, however, largely driven by recent PA. Additional analyses revealed that adjusting for waist circumference resulted in the greatest reduction in the estimates, suggesting that associations are largely mediated by adiposity (see Web Table 4). Estimates were also attenuated after excluding men with stroke, coronary heart disease, and diabetes at the 20-year follow-up, but associations with insulin, lung function, waist circumference, IL-6, CRP, tPA antigen, and high-sensitivity cardiac troponin T remained significant (Web Table 5). Conclusions were not changed when analyses were weighted according to trajectory-group probabilities (data not shown).

**Table 3.** Adjusted Association^a^ Between 20-Year Subjectively Determined Physical Activity Trajectories and Cardiovascular Markers at 20-Year Follow-up From Logistic or Linear Regression Models in Men Without Preexisting CVD and Diabetes (*n* = 3,079, Aged 58–81 Years at Follow-up), British Regional Heart Study, 1978–2000

Outcome Measure	Physical Activity Trajectory Group^b,c^													
	0-1-1		1-0-1		0-0-1		1-1-0		0-1-0		1-0-0		0-0-0	
	(*n* = 394; 12.8%)		(*n* = 198; 6.4%)		(*n* = 161; 5.2%)		(*n* = 270; 8.8%)		(*n* = 182; 5.9%)		(*n* = 221; 7.2%)		(*n* = 308; 10.0%)	
	B Coefficient	95% CI	B Coefficient	95% CI	B Coefficient	95% CI	B Coefficient	95% CI	B Coefficient	95% CI	B Coefficient	95% CI	B Coefficient	95% CI
Metabolic markers														
HDL, mmol/L^d^	−0.02	−0.06, 0.01	0.03	−0.02, 0.08	−0.01	−0.06, 0.04	−0.02	−0.06, 0.02	0.02	−0.03, 0.07	0.03	−0.01, 0.08	−0.03	−0.06, 0.01
LDL, mmol/L^d^	0.05	−0.05, 0.16	0.04	−0.10, 0.18	0.02	−0.14, 0.17	0.00	−0.13, 0.12	−0.14	−0.29, 0.01	−0.03	−0.16, 0.11	−0.06	−0.18, 0.06
Total cholesterol, mmol/L^d^	0.10	−0.02, 0.21	0.06	−0.09, 0.22	−0.01	−0.18, 0.17	0.01	−0.13, 0.14	−0.14	−0.31, 0.02	0.01	−0.14, 0.16	0.01	−0.12, 0.14
Triglycerides, mmol/L^d^	0.07	0.02, 0.12	0.00	−0.06, 0.07	−0.02	−0.09, 0.05	0.03	−0.03, 0.09	−0.02	−0.08, 0.05	−0.02	−0.08, 0.05	0.10	0.05, 0.15
Hypertension^e^	1.15	0.91, 1.46	1.43	1.05, 1.95	1.27	0.90, 1.79	1.44	1.09, 1.89	1.27	0.91, 1.76	1.40	1.03, 1.89	1.52	1.17, 1.98
Glucose, mmol/L^d^	0.01	−0.01, 0.03	0.01	−0.02, 0.04	0.03	0.00, 0.06	0.02	0.00, 0.04	0.05	0.02, 0.08	0.01	−0.02, 0.03	0.03	0.01, 0.05
Insulin, μ/mL^d^	0.06	0.00, 0.12	0.09	0.01, 0.17	0.04	−0.05, 0.13	0.07	0.00, 0.14	0.09	0.01, 0.18	0.02	−0.05, 0.09	0.16	0.09, 0.22
HbA1C, mmol/L^d,f^	0.00	−0.01, 0.02	0.00	−0.02, 0.02	0.01	−0.01, 0.04	0.00	−0.02, 0.02	0.02	−0.01, 0.04	0.00	−0.02, 0.03	0.02	0.00, 0.04
FEV_1_, L^d,g^	1.20	−5.39, 7.79	−5.15	−13.90, 3.59	−4.83	−14.47, 4.82	−12.19	−19.88, −4.50	−15.15	−24.28, −6.01	−12.63	−21.05, −4.20	−11.72	−19.09, −4.36
Waist circumference, cm^d^	0.55	−0.57, 1.67	1.15	−0.33, 2.64	2.84	1.20, 4.47	1.24	−0.06, 2.55	2.07	0.52, 3.62	4.20	2.77, 5.62	3.27	2.03, 4.52
Inflammatory/hemostatic markers														
IL-6, pg/ml^d,f^	0.03	−0.04, 0.10	−0.07	−0.17, 0.02	−0.01	−0.11, 0.10	0.16	0.08, 0.24	0.11	0.01, 0.21	0.13	0.04, 0.22	0.13	0.05, 0.21
CRP, mg/L^d,f^	−0.07	−0.18, 0.05	0.09	−0.07, 0.24	0.12	−0.05, 0.29	0.15	0.01, 0.28	0.08	−0.08, 0.24	0.29	0.14, 0.44	0.15	0.02, 0.28
Factor VIII, IU/dL^d,h^	0.80	−2.58, 4.18	−0.94	−5.42, 3.55	1.16	−3.79, 6.11	2.85	−1.09, 6.80	3.25	−1.44, 7.94	2.37	−1.95, 6.70	3.17	−0.61, 6.95
von Willebrand factor, IU/dL^d,h^	2.11	−2.81, 7.02	0.22	−6.30, 6.74	2.57	−4.62, 9.76	4.71	−1.02, 10.45	4.10	−2.71, 10.92	6.94	0.65, 13.22	3.46	−2.03, 8.96
tPA antigen, ng/mL^d,h^	0.19	−0.26, 0.64	0.23	−0.37, 0.82	−0.26	−0.92, 0.40	0.53	0.01, 1.06	0.92	0.30, 1.54	0.46	−0.12, 1.03	1.03	0.52, 1.53
D-dimer, ng/mL^d,f,h^	−0.04	−0.13, 0.04	−0.08	−0.19, 0.04	−0.10	−0.23, 0.02	0.09	−0.01, 0.19	0.07	−0.05, 0.19	0.12	0.02, 0.23	0.08	−0.02, 0.17
Cardiac markers														
Hs-TnT, pg/mL^d,f^	−0.03	−0.08, 0.02	0.02	−0.04, 0.09	−0.03	−0.11, 0.04	0.02	−0.04, 0.08	0.01	−0.06, 0.08	0.02	−0.05, 0.09	0.01	−0.04, 0.07
NT-proBNP, pg/mL^d,f,i^	−0.06	−0.18, 0.06	0.07	−0.10, 0.23	−0.02	−0.20, 0.16	0.14	0.00, 0.28	0.11	−0.06, 0.28	0.18	0.02, 0.34	−0.05	−0.19, 0.08

Abbreviations: CI, confidence interval; CRP, c-reactive protein; FEV_1_, forced expiratory volume in 1 second; HbA1C, glycated hemoglobin; HDL, high-density lipoprotein; Hs-TnT, high-sensitivity cardiac troponin T; IL-6, interleukin-6; LDL, low-density lipoprotein; NT-proBNP, *N*-terminal pro-brain natriuretic peptide; tPA, tissue plasminogen activator.

^a^ All models adjusted for age, occupational class, region of residence, smoking status, alcohol consumption and waist circumference (where waist circumference was the outcome models adjusted for all other factors except waist circumference), and lipid-lowering medication.

^b^ At each of the 3 time points, men were classified as active (1) if they had at least “light” activity or inactive (0) for inactive or occasional activity. Men were allocated to 1 of 8 possible trajectories, which are indicated by combinations of zeros and ones (1, active; 0, inactive). For example, (0-0-0) represents inactivity at all periods, while (1-0-0) indicates being active at baseline only.

^c^ The 1-1-1 group (persistently active; *n* = 1,345, 43.7%) served as the reference group.

^d^ Additionally adjusted for blood pressure-lowering medication.

^e^ Presented as an odds ratio and 95% confidence interval. Hypertension was defined as a systolic blood pressure of ≥160 mm Hg, diastolic blood pressure ≥100 mm Hg, or use of antihypertensive medication.

^f^ Log transformed.

^g^ FEV_1_ was standardized for height by multiplying FEV_1_ by the square of the mean population height (meters) divided by each participant’s height.

^h^ Additionally adjusted for warfarin.

^i^ Data were missing for an additional 178 men (*n* = 2,901).

## DISCUSSION

The present study found that the most active trajectory (moderate/increasing) from midlife to old age was most favorably associated with CVD biomarkers in old age. However, even more modest but persistent PA (light/stable) yielded significant health benefit. Sustained light volumes of PA into old age, including occasional sporting exercise or moderate amounts of walking combined with some gardening/do-it-yourself projects, may be sufficient to reduce a range of CVD risk factors.

To our knowledge, this is the first study to explore associations between trajectories of PA and CVD risk factors using GBTM. Our findings are not directly comparable with previous studies using more conventional approaches for identifying trajectories due to the different groups that we have identified using GBTM. Previous studies categorizing groups manually using a clinically meaningful cutpoint suggest that individuals who become active in old age present a better CVD biomarker profile than those who remain or become inactive, including an improved lipid profile and glucose tolerance and lower levels of inflammatory and hemostatic markers and *N*-terminal pro-brain natriuretic peptide (7–11, 24). However, using GBTM, we did not observe groups of individuals who made clear shifts from low to high PA or vice versa. Instead our findings suggest that PA in old age is largely dictated by PA in midlife or earlier, indicating that early engagement is crucial for lowering CVD risk factors in old age. Generally, our findings are consistent with evidence that sustained higher levels of PA are associated with the most optimal CVD biomarker profile.

Moreover, due to the more subjective approaches that are often adopted for classifying trajectories, usually involving collapsing PA data into a small number of categories, the dose or volume of PA required across the life course to achieve benefit remains unclear. Given that few older adults consistently meet or start meeting current PA guidelines (7.9% and 4.9%, respectively) (25), it is important to understand whether lower doses of PA are sufficient in old age. Although the guidelines recommend that adults should spend 150 minutes per week doing moderate-to-vigorous physical activity (MVPA), evidence that lower doses might be sufficient to achieve health benefits has been building (26). Previous results from this cohort using self-reported measures of PA have shown that light amounts of self-reported PA measured at a single time point were associated with lower levels of inflammatory and hemostatic markers, *N*-terminal pro-brain natriuretic peptide, and risk of coronary heart disease (10). The present study builds on this evidence by showing that sustaining a light dose of PA from midlife to old age can be sufficient to achieve such benefits, which is perhaps a more feasible target for inactive middle-aged and older adults. There is also accumulating evidence that lower intensities of activity might be sufficient to achieve benefit. Indeed, it has been shown that device-measured light-intensity PA is associated with lower levels of cardiac markers and reduced mortality risks, independent of MVPA (27–29).

In models that included both the trajectory and current PA, the trajectory remained significantly associated with the majority of the metabolic markers. Long-term persistent PA also proved optimal for metabolic outcomes when trajectories were classified using a conventional approach. Together these results suggest that sustained PA may have cumulative benefits to metabolic health, making it clinically important to consider PA history. These findings are consistent with a 5-year prospective study in older US adults that found that consistently meeting PA guidelines through walking was associated with the lowest risk of developing metabolic risk factors (24). One important pathway that could explain the metabolic responses to chronic PA is changes to body composition. Regular sustained PA is associated with reductions in visceral fat, increases in skeletal muscle mass, and improved metabolic efficiency of muscle (30).

In contrast to the metabolic markers, associations with inflammatory and hemostatic biomarkers were largely driven by recent PA rather than long-term engagement. Intervention studies have also demonstrated that reductions in inflammation can be achieved from fairly short-term increases in PA (31). Observations from the 1946 British Birth Cohort Study suggest that more frequent participation in leisure-time PA across adulthood (ages 36–64 years) is associated with lower CRP, IL-6, and tPA antigen in men at ages 60–64 years (11). Our data suggest that such benefits might be achieved by sustaining light volumes of PA across adulthood; however, the cumulative effect of PA across the life course might be more important for preserving metabolic health than for reducing inflammation and endothelial dysfunction. Although both of these studies are in older British men, they are not directly comparable given the differences in how PA and trajectories of PA were defined. Additional prospective studies with repeated measures of PA are required to fully understand whether the effects of PA on biomarkers are due to long-term engagement or participation at specific life stages.

Many of the observed associations were substantially, but not completely, attenuated by adiposity. Adiposity is an important pathway explaining the relationship between PA and CVD biomarkers, but there are other pathways involved. Other studies have also shown that associations between PA and CVD risk factors persist after controlling for adiposity (7, 9, 11). Associations were also largely attenuated when men who developed CVD over the 20-year follow-up were excluded, but many remained significant, strengthening the likelihood of a causal link between long-term PA from midlife and CVD risk factors in old age.

The key strength of this study is the long-term follow-up, including repeated PA measurements over an understudied period of the life course. Follow-up from midlife, a period before many risk factors for CVD present themselves, reduces the possibility of reverse causation. Unfortunately, we were unable to capture early-life PA, which may already have influenced CVD risk factors in later life. Another strength is the data-driven approach to identify trajectories. This enabled us to identify the unobserved patterns of PA, which could not have been described using conventional methods, and more precisely determine the optimal long-term patterns of PA for CVD risk. Another advantage of GBTM over conventional methods is that it can incorporate time-stable and time-varying covariates in the modeling process, allowing the shapes of each trajectory to vary according to variables beyond age or time. This results in more accurate descriptions of the trajectories and thus more reliable estimates of the associations with outcomes of interest.

A notable limitation is the use of self-reported PA, which is prone to recall bias. Device-measured PA may be more sensitive to changes in PA and more accurately estimate the volume and intensity required to reduce CVD risk factors (32). Nevertheless, the PA score has been validated against heart rate, forced expiratory volume in 1 second, and device-measured PA (22). In addition, it is possible that the early development of chronic conditions, before midlife, may have already influenced PA trajectories; thus we cannot rule out reverse causation. Further, subject attrition may have biased our results given that men who dropped out of the study were less active and more likely to have a range of health conditions at baseline. However, we previously showed that these trajectories are comparable when men with only 1 PA measurement were also included (14). Finally, it is important to note that our findings are only in men and may not be generalizable to women and nonwhite ethnic groups.

In conclusion, trajectories of PA from midlife to old age were associated with established and novel CVD risk factors. Although following a moderate/increasing trajectory proved optimal, a light/stable trajectory yielded similar benefits. This supports the argument that even fairly modest but sustained volumes of PA into old age may protect against CVD. This is important given that higher doses and intensities of PA are more challenging in aging adults.

## Supplementary Material

Web Material

## Abbreviations

BMI: body mass index
CRP: C-reactive protein
CVD: cardiovascular disease
GBTM: group-based trajectory modeling
IL-6: interleukin-6
PA: physical activity
tPA: tissue plasminogen activator.
