# Supplementary material for: Association Between 20-Year Trajectories of Nonoccupational Physical Activity From Midlife to Old Age and Biomarkers of Cardiovascular Disease: A 20-Year Longitudinal Study of British Men
Source: Am J Epidemiol. 2018 Aug 14;187(11):2315–23. doi: 10.1093/aje/kwy157 (PMC6211233; doi:10.1093/aje/kwy157)
Supplement: Web Material [file kwy157aggiowebmaterialfinal.pdf]

Web Material

Association Between 20-Year Trajectories of Nonoccupational Physical Activity From Midlife to Old Age and Biomarkers of Cardiovascular Disease: A 20-Year Longitudinal Study of British Men

Daniel Aggio, Efstathios Papachristou, Olia Papacosta, Lucy T. Lennon, Sarah Ash, Peter H. Whincup, S. Goya Wannamethee, and Barbara J. Jefferis

Web Appendix 1: Cardiovascular Disease Biomarkers & Measures

Web Figure 1: Recruitment Flow Chart

Web Table 1. Model Search Process for Physical Activity Trajectories (n=3,331)

Web Table 2. Determining the Highest Model Function of the 3 Physical Activity Trajectory Groups (n=3,331)

Web Table 3. Association (B, 95% CI) Between 20-Year Physical Activity Trajectories and Cardiovascular Markers at 20-Year Follow Up from Linear Regression Models, Adjusting for Current PA Score (N=3,258)

Web Table 4. Association (B, 95% CI) Between 20-Year Physical Activity Trajectories and Cardiovascular Markers at 20-Year Follow Up from Linear Regression Models, Without Adjustment for Adiposity

Web Table 5. Adjusted Association (B, 95% CI) Between 20-Year Physical Activity Trajectories and Cardiovascular Markers at 20-Year Follow Up from Linear Regression Models Excluding Men with CVD and Diabetes at 20-Year Follow Up (N=2,691)

Web appendix 1

Cardiovascular Disease Biomarkers & Measures

A fasting blood sample was collected using the Sarstedt Monovette system. Total, HDL cholesterol and triglyceride were measured using a Hitachi 747 automated analyser (Hitachi, Tokyo, Japan). Total and HDL cholesterol were determined using methods described by Siedel et al., (18) and Sugiuchi et al., (19) and low-density lipoprotein was calculated using the Friedrickson-Friedwald equation. Plasma glucose was measured using a glucose oxidase method (Falcor 600 automated analyser, A. Menarini Diagnostics, Wokingham, UK). Serum insulin was measured using an enzyme-linked immunosorbent assay (ELISA) that does not cross-react with proinsulin.(20) Hemoglobin A1c was determined using a Drew Hb Gold HPLC analyser (Drew Scientific Group Plc, Barrow in Furness, UK). Blood was anticoagulated with 0.109 mol/L trisodium citrate (9:1 vol:vol) for measurement of coagulation factor VIII in an MDA-180 coagulometer (Organon Teknika, Cambridge, UK). Plasma levels of tissue plasminogen activator antigen and D-dimer were measured with ELISAs (Biopool AB, Umea, Sweden), as was von Willebrand factor antigen (DAKO, High Wycombe, UK). C-reactive protein was assayed by ultrasensitive nephelometry (Dade Behring, Milton Keynes, UK). Interleukin-6 (IL-6) was assayed using a high-sensitivity ELISA (R&D Systems). N-terminal pro-brain natriuretic peptide was measured using the Elecsys 2010 (Roche Diagnostics, Burgess Hill, UK).(21) Cardiac troponin T was measured using a high-sensitivity method on an e411 analyser (Roche Diagnostics, Burgess Hill, UK).

Height was measured to the nearest 0.1cm using a stadiometer (Holtain, Crosswell, United Kingdom). Weight was measured in light clothing to the last complete 0.1kg using a digital electronic scale (Soehnle-Waagen, Murrhardt, Germany). Waist circumference was taken from the midpoint between the iliac crest and lower ribs and was measured using an insertion tape (CMS Ltd, London,

United Kingdom). Forced Expiratory Volume in one second (FEV_1) was measured using a Vitalograph Compact spirometer. Systolic and diastolic blood pressure were each measured twice with the subject seated, using a Dinamap 1846SX blood pressure recorder and the mean of two readings was used.

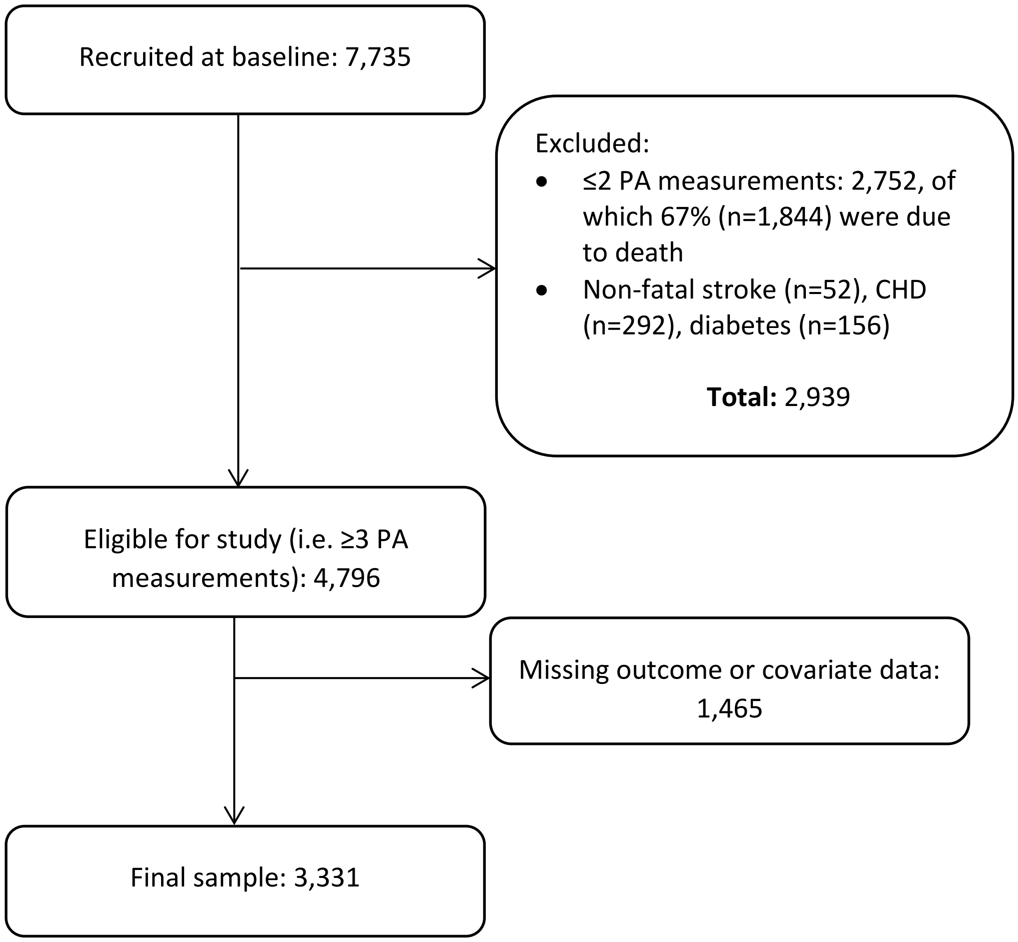

Web Figure 1. Recruitment Flow Chart, British Regional Heart Study, 1978-2000

Web Table 1. Model Search Process for Physical Activity Trajectories (n=3,331), British Regional Heart Study, 1978-2000

Number of groups	BIC	Log Bayes Factor (2*ΔBIC)	Estimated group %	Actual group %	Posterior probability
			64.5	65.0	0.95
2	-21645.4		35.5	35.0	0.93
			23.7	21.8	0.81
			49.5	51.5	0.83
3	-21483.3	324.2	26.8	26.7	0.91
			13.6	11.2	0.81
			50.0	53.0	0.85
			29.4	28.9	0.86
4	-21454.3	58.0	7.1	7.0	0.85
			3.3	3.5	0.78
			14.3	11.5	0.72
			46.9	49.9	0.82
			28.6	28.3	0.86
5	-21521.5	-134.4	7.0	6.8	0.85

BIC, Bayesian information criterion.

Models adjusted for employment status and number of cardiovascular disease diagnoses as time-varying covariates, and occupational class, marital status, number of children, region, body mass index , arthritis, bronchitis, blood pressure, breathlessness, chest pain, smoking status, alcohol consumption and breakfast consumption at baseline.

Web Table 2. Determining the Highest Model Function of the 3 Physical Activity Trajectory Groups (n=3,331), British Regional Heart Study, 1978-2000 [ab]

	**1st iteration**		**2nd iteration**			
**Group**	**Highest function**	***p***	**Highest function**	***p***	**Final estimated group %**	**Final actual group %**
Group 1	Quadratic	<0.001	Quadratic	<0.001	23.3	21.3
Group 2	Quadratic	0.795	Linear	0.008	49.8	51.8
Group 3	Quadratic	0.233	Linear	<0.001	26.9	27.0

[a] Starting with quadratic, the level of the polynomial function for each group was reduced at each iteration until each parameter estimate was statistically significant ($p<0.05$). [b] Models adjusted for employment status and number of cardiovascular disease diagnoses as time-varying covariates, and occupational class, marital status, number of children, region, body mass index, arthritis, bronchitis, blood pressure, breathlessness, chest pain, smoking status, alcohol consumption and breakfast consumption at baseline.

Web Table 3. Adjusted Association[a] (B, 95% CI) Between 20-Year Physical Activity Trajectories and Cardiovascular Markers at 20-Year Follow up from Linear or Logistic Regression Models[b], Adjusting for Current PA Score (N=3,258), British Regional Heart Study, 1978-2000

	PHYSICAL ACTIVITY TRAJECTORY GROUPS				
**Outcome measure**	**Light Stable**		**Moderate Increasing**		
	B coefficient	95% CI	B coefficient	95% CI	p trend
**Metabolic markers**					
HDL, mmol/L [c]	-0.03	-0.07, 0.01	0.01	-0.04, 0.06	0.719
LDL, mmol/L [c]	0.06	-0.05, 0.17	0.01	-0.14, 0.16	0.917
Total cholesterol, mmol/L [c]	0.02	-0.11, 0.14	-0.07	-0.24, 0.10	0.428
Triglycerides, mmol/L [c]	-0.02	-0.08, 0.03	-0.09	-0.16, -0.02	0.015
Hypertension [d]	0.84	0.65, 1.07	0.73	0.52, 1.03	-
Glucose, mmol/L [c]	-0.03	-0.05, -0.01	-0.04	-0.07, -0.01	0.008
Insulin, µ/mL [c]	-0.06	-0.13, 0.00	-0.07	-0.16, 0.01	0.102
HbA1C, mmols/l [ce]	-0.02	-0.04, -0.01	-0.04	-0.06, -0.01	0.002
FEV_1, L [cf]	10.15	3.27, 17.03	10.20	0.68, 19.72	0.039
Waist circumference (cm) [c]	-2.47	-3.64, -1.30	-2.18	-3.81, -0.56	0.010
**Inflammatory/hemostatic markers**					
IL-6, pg/ml [ce]	-0.05	-0.12, 0.03	-0.04	-0.14, 0.07	0.504
CRP, mg/L [ce]	-0.12	-0.24, 0.00	-0.12	-0.29, 0.04	0.155

Factor VIII, IU/dL [cg]	-4.09	-7.63, -0.55	-5.28	-10.18, -0.38	0.036
vWF, IU/dL [cg]	-2.76	-7.91, 2.40	-4.77	-11.90, 2.37	0.191
tPA, ng/mL [cg]	-0.28	-0.75, 0.19	-0.67	-1.32, -0.01	0.045
d-dimer, ng/mL [ceg]	-0.03	-0.12, 0.06	0.03	-0.09, 0.16	0.521
**Cardiac markers**					
Hs-TnT, pg/mL [ce]	-0.05	-0.11, 0.00	-0.02	-0.10, 0.05	0.586
NT-proBNP, pg/mL [ceh]	0.04	-0.09, 0.17	0.13	-0.05, 0.30	0.158

Abbreviations: CI, confidence interval; OR, odds ratio; HDL, high-density lipoprotein; LDL, low-density lipoprotein; HbA1C, hemoglobin A1c; FEV_1, forced expiratory volume in 1 second; IL-6, interleukin-6; CRP, c-reactive protein; vWF, von Willebrand factor; tPA, tissue plasminogen activator antigen; Hs-TnT, high-sensitivity cardiac troponin T; NT-proBNP, n-terminal pro-brain natriuretic peptide

[a]All models adjusted for age, occupational class, region of residence, smoking status, alcohol consumption and waist circumference (where waist circumference was the outcome models adjusted for all other factors except waist circumference), lipid-lowering medication and current physical activity score at 20-year follow up

[b]Low decreasing group served as the reference group

[c] additionally adjusted for blood pressure-lowering medication

[d] presented as an odds ratio and 95% confidence interval. Hypertension, high systolic (≥160 mmHg) or diastolic (≥100 mmHg) blood pressure or taking anti-hypertensive medication

[e] log transformed

[f] FEV_1 was standardised for height by multiplying FEV_1 by the square of the mean population height (metres) divided by each participant's height.

[g] additionally adjusted for warfarin

[h] data were missing for an additional 190 men (n=3,068)

Web Table 4. Adjusted Association[a] (B, 95% CI) Between 20-Year Physical Activity Trajectories and Cardiovascular Markers at 20-Year Follow up from Linear or Logistic Regression Models[b], Without Adjustment for Adiposity, British Regional Heart Study, 1978-2000

	**PHYSICAL ACTIVITY TRAJECTORY GROUPS**				
**Outcome measure**	**Light Stable**		**Moderate Increasing**		
	B coefficient	95% CI	B coefficient	95% CI	p trend
**Metabolic markers**					
HDL, mmol/L [c]	0.00	-0.03, 0.03	0.05	0.02, 0.09	0.001
LDL, mmol/L [c]	0.08	0.00, 0.17	0.03	-0.07, 0.13	0.636
Total cholesterol, mmol/L [c]	0.03	-0.06, 0.12	-0.02	-0.13, 0.09	0.627
Triglycerides, mmol/L [c]	-0.07	-0.11, -0.03	-0.12	-0.17, -0.08	<0.001
Hypertension [d]	0.71	0.59, 0.85	0.58	0.47, 0.72	-
Glucose, mmol/L [c]	-0.03	-0.05, -0.01	-0.04	-0.06, -0.02	<0.001
Insulin, µ/mL [c]	-0.17	-0.23, -0.12	-0.23	-0.29, -0.17	<0.001
HbA1C, mmols/l [ce]	-0.03	-0.04, -0.01	-0.03	-0.04, -0.01	0.001
FEV_1, L [cf]	15.33	10.08, 20.58	24.05	17.97, 30.13	<0.001
Waist circumference (cm) [c]	-3.25	-4.13, -2.36	-4.22	-5.24, -3.19	<0.001
**Inflammatory/hemostatic markers**					
IL-6, pg/ml [ce]	-0.15	-0.21, -0.10	-0.22	-0.28, -0.15	<0.001

CRP, mg/L [ce]	-0.28	-0.37, -0.18	-0.37	-0.48, -0.26	<0.001
Factor VIII, IU/dL [cg]	-5.84	-8.54, -3.15	-6.80	-9.92, -3.68	<0.001
vWF, IU/dL [cg]	-6.64	-10.54, -2.74	-7.61	-12.12, -3.10	0.002
tPA, ng/mL [cg]	-1.07	-1.45, -0.70	-1.55	-1.98, -1.12	<0.001
d-dimer, ng/mL [ceg]	-0.11	-0.17, -0.04	-0.12	-0.20, -0.04	0.004
**Cardiac markers**					
Hs-TnT, pg/mL [ce]	-0.09	-0.13, -0.05	-0.05	-0.10, -0.01	0.050
NT-proBNP, pg/mL [ceh]	-0.06	-0.15, 0.04	-0.04	-0.15, 0.08	0.580

Abbreviations: CI, confidence interval; OR, odds ratio; HDL, high-density lipoprotein; LDL, low-density lipoprotein; HbA1C, hemoglobin A1c; FEV_1, forced expiratory volume in 1 second; IL-6, interleukin-6; CRP, c-reactive protein; vWF, von Willebrand factor; tPA, tissue plasminogen activator antigen; Hs-TnT, high-sensitivity cardiac troponin T; NT-proBNP, n-terminal pro-brain natriuretic peptide

[a]All models adjusted for age, occupational class, region of residence, smoking status, alcohol consumption and lipid-lowering medication

[b]Low decreasing group served as the reference group

[c] additionally adjusted for blood pressure-lowering medication

[d] presented as an odds ratio and 95% confidence interval. Hypertension, high systolic (≥160 mmHg) or diastolic (≥100 mmHg) blood pressure or taking anti-hypertensive medication

[e] log transformed

[f] FEV_1 was standardised for height by multiplying FEV_1 by the square of the mean population height (metres) divided by each participant's height.

[g] additionally adjusted for warfarin

[h] Data were missing for an additional 194 men (n=3,137)

Web Table 5. Adjusted Association[a] (B, 95% CI) Between 20-Year Physical Activity Trajectories and Cardiovascular Markers at 20-Year Follow Up from Linear or Logistic Regression Models[b] Excluding Men with CVD and Diabetes at 20-Year Follow Up (N=2,691), British Regional Heart Study, 1978-2000

	PHYSICAL ACTIVITY TRAJECTORY GROUPS				
**Outcome measure**	**Light stable**		**Moderate increasing**		
	B coefficient	95% CI	B coefficient	95% CI	p trend
**Metabolic markers**					
HDL, mmol/L [c]	-0.03	-0.06, 0.01	0.01	-0.03, 0.05	0.443
LDL, mmol/L [c]	0.09	-0.01, 0.19	0.04	-0.07, 0.15	0.597
Total cholesterol, mmol/L [c]	0.07	-0.04, 0.18	0.03	-0.10, 0.15	0.835
Triglycerides, mmol/L [c]	0.00	-0.04, 0.05	-0.03	-0.08, 0.02	0.192
Hypertension [d]	0.80	0.65, 0.99	0.72	0.57, 0.92	-
Glucose, mmol/L [c]	0.00	-0.01, 0.01	0.00	-0.01, 0.01	0.694
Insulin, µ/mL [c]	-0.05	-0.10, 0.01	-0.09	-0.15, -0.03	0.002
HbA1C, mmols/l [ce]	-0.01	-0.02, 0.01	-0.01	-0.02, 0.01	0.270
FEV_1, L [cf]	12.22	6.13, 18.30	21.06	14.11, 28.01	<0.001
Waist circumference (cm) [c]	-3.01	-3.99, -2.02	-4.18	-5.31, -3.05	<0.001
**Inflammatory/hemostatic markers**					

IL-6, pg/ml [ce]	-0.14	-0.20, -0.08	-0.20	-0.27, -0.13	<0.001
CRP, mg/L [ce]	-0.19	-0.30, -0.09	-0.28	-0.40, -0.16	<0.001
Factor VIII, IU/dL [cg]	-3.04	-6.10, 0.01	-4.04	-7.53, -0.06	0.030
vWF, IU/dL [cg]	-4.62	-8.96, -0.28	-4.88	-9.84, 0.08	0.078
tPA, ng/mL [cg]	-0.63	-1.02, -0.24	-0.89	-1.34, -0.44	<0.001
d-dimer, ng/mL [ceg]	-0.10	-0.17, -0.02	-0.11	-0.20, -0.03	0.017
**Cardiac markers**					
Hs-TnT, pg/mL [ce]	-0.06	-0.11, -0.02	-0.05	-0.10, 0.01	0.161
NT-proBNP, pg/mL [ceh]	-0.10	-0.16, 0.05	-0.08	-0.20, 0.05	0.235

Abbreviations: CI, confidence interval; OR, odds ratio; HDL, high-density lipoprotein; LDL, low-density lipoprotein; HbA1C, hemoglobin A1c; FEV_1, forced expiratory volume in 1 second; IL-6, interleukin-6; CRP, c-reactive protein; vWF, von Willebrand factor; tPA, tissue plasminogen activator antigen; Hs-TnT, high-sensitivity cardiac troponin T; NT-proBNP, n-terminal pro-brain natriuretic peptide

[a]All models adjusted for age, occupational class, region of residence, smoking status, alcohol consumption and waist circumference (where waist circumference was the outcome models adjusted for all other factors except waist circumference) and lipid-lowering medication

[b]Low decreasing group served as the reference group

[c] additionally adjusted for blood pressure-lowering medication

[d] presented as an odds ratio and 95% confidence interval. Hypertension, high systolic (≥160 mmHg) or diastolic (≥100 mmHg) blood pressure or taking anti-hypertensive medication

[e] log transformed

[f] FEV_1 was standardised for height by multiplying FEV_1 by the square of the mean population height (metres) divided by each participant's height.

[g] additionally adjusted for warfarin

[h] data were missing for an additional 159 men (n=2,532)
